# Effect of lipopolysaccharide (LPS) and peptidoglycan (PGN) on human mast cell numbers, cytokine production, and protease composition

**DOI:** 10.1186/1471-2172-9-45

**Published:** 2008-08-07

**Authors:** Arnold S Kirshenbaum, Emily Swindle, Marianna Kulka, Yalin Wu, Dean D Metcalfe

**Affiliations:** 1 Laboratory of Allergic Diseases, National Institute of Allergy and Infectious Diseases, National Institutes of Health, Bethesda, MD 20892, USA; 2 Institute for Nutrisciences and Health, NRC Canada, 550 University Avenue, Charlottetown, PE, C1A 4P3, Canada

## Abstract

**Background:**

Human mast cell (HuMC) maturation occurs in tissues interfacing with the external environment, exposing both mast cell progenitors and mature mast cells, to bacteria and their products. It is unknown, however, whether long- or short-term exposure to bacteria-derived toll-like receptor (TLR) ligands, such as lipopolysaccharide (LPS) or peptidoglycan (PGN), influences HuMC biology.

**Results:**

Over 6 wks of culture, LPS had minimal effect on HuMC numbers but increased CD117, tryptase and chymase expression. PGN inhibited HuMC development. For mature mast cells, LPS in the presence of rhSCF (10 ng/ml) increased CD117, tryptase, chymase and carboxypeptidase expression, primarily in CD117^low ^HuMC. LPS decreased FcεRI expression and β-hexosaminidase release; but had no effect on LTC_4 _and PGD_2 _production. PGN reduced HuMC numbers; and CD117 and tryptase expression. IL-1β and IL-6 (in addition to IL-8 and IL-12) were detected in short-term culture supernatants of LPS treated cells, and reproduced the increases in CD117, tryptase, chymase, and carboxypeptidase expression observed in the presence of LPS. Comparative studies with mouse bone marrow-derived mast cells from wild type, but not TLR4 knockout mice, showed increases in mRNA of mouse mast cell chymases MMCP-1, MMCP-2 and MMCP-4.

**Conclusion:**

PGN inhibits HuMC growth, while LPS exerts its primary effects on mature HuMC by altering cytokine production and protease composition, particularly at low concentrations of SCF. These data demonstrate the ability of bacterial products to alter HuMC mediator production, granular content, and number which may be particularly relevant at mucosal sites where HuMC are exposed to these products.

## Background

Human mast cells (HuMC) originate from bone marrow-derived CD34^+ ^pluripotent progenitor cells [1] and migrate as immature cells from the bone marrow to tissue sites including the lung and gastrointestinal tract. There they mature and participate in both innate and acquired immune responses with production of cytokines and other inflammatory mediators [2]. Mast cell growth and development thus may occur in a tissue that interfaces with the external environment, potentially exposing HuMC during their development to bacterial products which could have an impact on their subsequent behavior. Consistent with this idea is the observation that HuMC have been shown to express Toll-like receptors (TLR) 1–7, and 9 both *in vitro *and *in vivo *(lung) [3]; and exposure to such bacterial products as endotoxin (lipopolysaccharide; LPS) or peptidoglycan (PGN) leads to expression and release of TNF-α, GM-CSF, IL-1, IL-5, IL-10, IL-13 and IL-15 [4-6]. However, and in a related question, it is currently not known whether bacteria-derived products alter the growth and development of HuMC.

We therefore asked whether long or short-term exposure to LPS and PGN could alter specific HuMC characteristics including growth; surface FcεRI and CD117 expression; and β-hexosaminidase, LTC_4 _and PGD_2 _release; protease expression and composition, and cytokine release. As will be shown, LPS augmented protease expression and altered protease composition in developing and mature HuMC, while reducing FcεRI expression and IgE-mediated degranulation. This effect in short-term cultures was mediated through HuMC release of IL-1β and IL-6. PGN inhibited HuMC growth at all concentrations studied.

## Results

### Effect of LPS and PGN on progenitor HuMC in 6 wk (long-term) cultures

**Figure 1:**
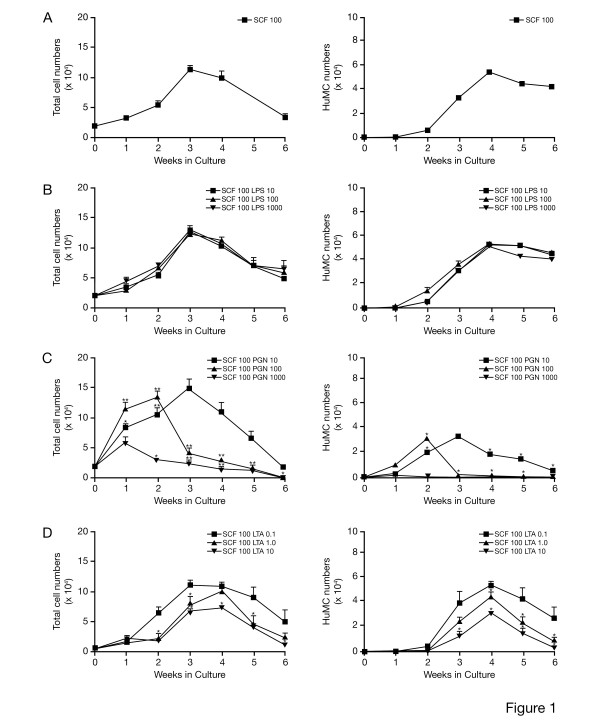
**Cultures of CD34^+ ^derived HuMC over 6 wks in the presence of rhSCF alone, or with LPS, PGN or LTA**. (A-D): Total cell numbers – left; HuMC numbers- right. A) 100 ng/ml rhSCF; B) 100 ng/ml rhSCF and 10–1000 ng/ml LPS; C) 100 ng/ml rhSCF and 10–1000 μg/ml PGN. Results are shown as the mean ± SEM of 3 separate experiments performed in duplicate; D) 100 ng/ml rhSCF and 0.1–10 μg/ml LTA. Results are shown as the mean ± SEM of 2 separate experiments performed in triplicate.

To determine the effect of LPS or PGN over 6 wks on HuMC growth and development, 10–1000 ng/ml LPS or 10–1000 μg/ml PGN was added to CD34+ cultures containing 100 ng/ml rhSCF. Kimura's staining of metachromatic positive HuMC correlated with tryptase staining of HuMC granules on cytopreparations, did not differ by greater than 5%, and was used for counting small numbers of HuMCs available to study, with minimal cell loss. As shown in Figure 1A, incubation of CD34+ cells with SCF alone resulted in increased total cell numbers by 3 wks which then gradually declined by 6 wks, while HuMC numbers rose steadily from 2–4 wks and were sustained up to 6 wks, by which time all cells in culture were HuMC. The addition of LPS to CD34^+ ^cells over 6 wks of culture had no effect on total cell or HuMC numbers (Fig. 1B). In contrast, PGN initially increased total cell numbers by 1–3 wks, but this increase was short lived, and total numbers rapidly declined after 2–3 wks (Fig. 1C). HuMC growth was totally suppressed by PGN at 1000 μg/ml while PGN at 100 μg/ml suppressed HuMC growth by 3 wks. HuMC were seen in culture in the presence of 10 μg/ml PGN, but numbers were less than half of numbers seen with rhSCF alone (Fig. 1C; compare with 1A). Inhibition of HuMC growth was also seen with another TLR2 agonist, lipoteichoic acid (LTA). As seen in Figure 1D, LTA at 1.0–10 μg/ml decreased total cell numbers starting at 2–3 wks. The addition of LTA at 1.0–10 μg/ml suppressed HuMC growth beginning at 3–4 wks and for the duration of the cultures (Fig. 1D; compare with 1A). Thus, the addition of LPS over 6 wks did not alter HuMC numbers while PGN and LTA inhibited HuMC outgrowth.

### Effect of LPS and PGN on 8 wk old HuMC CD117 and FcεRI expression in 72 hr (short-term) cultures

**Figure 2:**
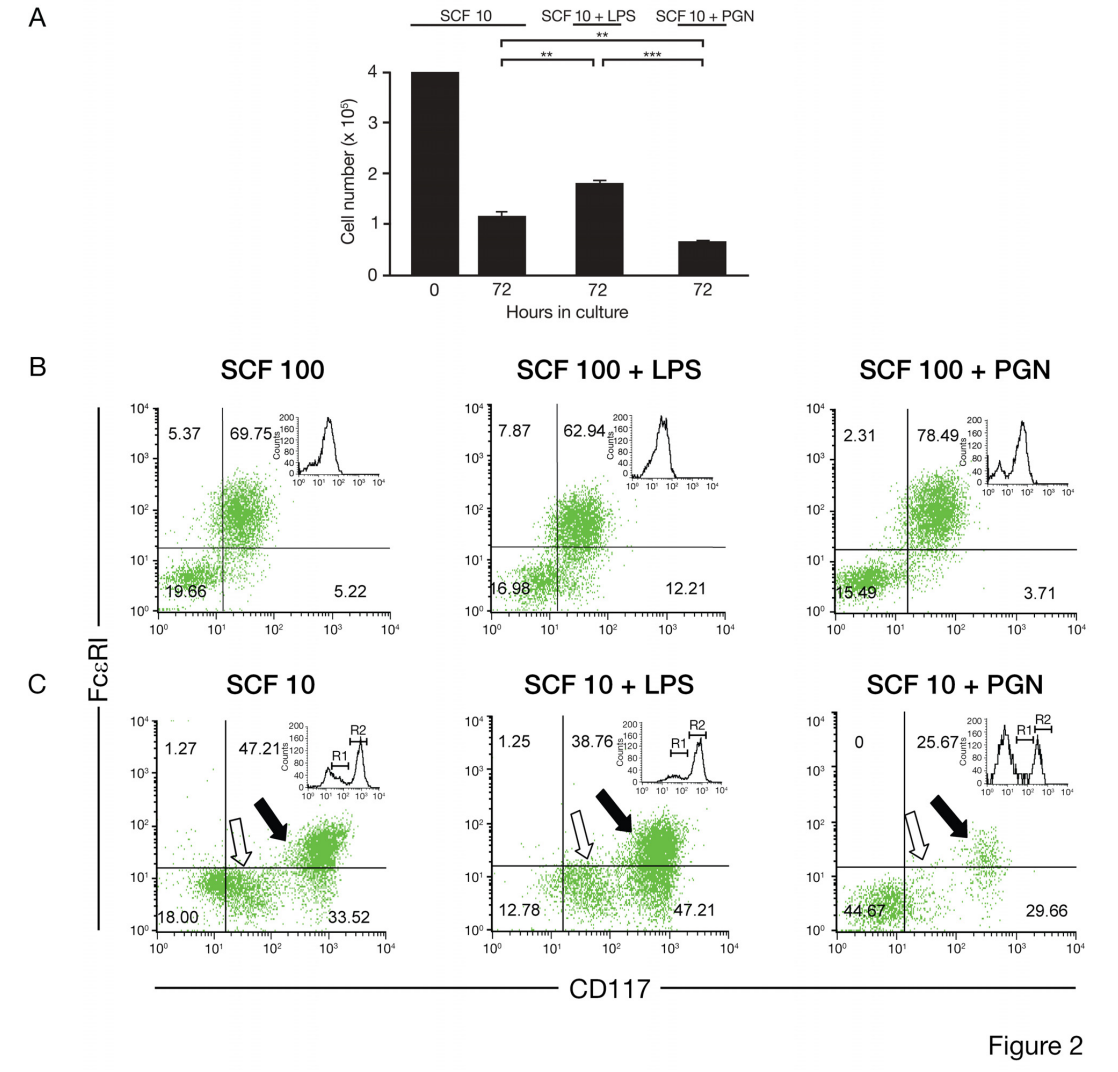
**FACS analysis of 8 wk FcεRI^+^/CD117^+ ^HuMC cultured in rhSCF alone, with LPS or PGN**. A) HuMC numbers following culture for 72 hr in 10 ng/ml rhSCF alone and with 100 ng/ml LPS or 10 μg/ml PGN. Results are shown as the means ± SEM of 3 separate experiments performed in duplicate; B and C) Representative dot plots of HuMC expression of FcεRI (vertical axis) and CD117 (horizontal axis) following culture for 72 hr in 100 ng/ml (B) or 10 ng/ml (C) rhSCF (left-side plots), rhSCF and 100 ng/ml LPS (middle plots), and rhSCF and 10 μg/ml PGN (right-side plots) In C, CD117^low ^(open arrows; insert R1 histogram) and CD117^high ^(solid arrows; insert R2 histogram) are labeled. Representative dot plots are shown of n = 3 separate experiments performed in duplicate.

We next reasoned that exposure to LPS or PGN would occur to more mature mast cells in some situations and promote HuMC differentiation. We thus cultured mature 8 wk old HuMC in either 100 ng/ml or 10 ng/ml rhSCF in the presence or absence of 100 ng/ml LPS or 10 μg/ml PGN (selected on the basis of long term culture results) for 72 hrs. Cells were then counted, harvested and examined by flow cytometry for FcεRI and CD117 expression. LPS 100 ng/ml or 10 ng/ml PGN had no effect on HuMC numbers when cultured in combination with rhSCF 100 ng/ml for 72 hr. When rhSCF was reduced to 10 ng/ml (Fig. 2A), HuMC numbers decreased consistent with less rhSCF in the cultures. Viability of the remaining HuMC after 72 hrs ranged from 90–95%. The added presence of LPS increased HuMC numbers after 72 hrs as compared with rhSCF alone (n = 3, p < 0.01), while PGN again resulted in fewer HuMC in culture (n = 3, p < 0.01). The addition of either 10 μg/ml PGN or 10 μg/ml LTA caused an increase of approximately 27% and 55%, respectively, of apoptotic cells compared to cultures in 10 ng/ml rhSCF alone, in contrast to 100 ng/ml LPS which reduced apoptosis by approximately 50%. To eliminate cell debris or any monocytes from being analyzed, CD117^+^/FcεRI^+ ^HuMC were gated, followed by examination for other surface markers or intracellular proteases. As shown in Figure 2B, in the presence of 100 ng/ml rhSCF, a homogeneous population of CD117^+^/FcεRI^+ ^HuMC was seen (approximately 70%). In the added presence of 100 ng/ml LPS, CD117^+^/FcεRI^+ ^cells decreased by approximately 7% for HuMC, with a concurrent decrease in FcεRI expression (right lower quadrant). Reducing the concentration of rhSCF to 10 ng/ml for 72 hrs (Fig. 2C) gave rise to two distinct HuMC CD117^+ ^subpopulations: CD117^low ^(open arrows) and CD117^high ^(solid arrows), with a log-fold increase in MFI for CD117^high ^cells as compared with cultures using 100 ng/ml rhSCF alone (Fig. 2C inserts). The addition of 100 ng/ml LPS over 72 hrs to CD117^low ^and CD117^high ^HuMC maintained in 10 ng/ml rhSCF decreased FcεRI expression on CD117^+^/FcεRI^+ ^cells (right lower quadrant) by approximately 9%. Histochemical examination of CD117^high ^and CD117^low ^cells confirmed that both populations shared characteristics, such as numerous surface projections, large and irregular nuclei, and abundant cytoplasmic granules, consistent with cultured HuMC, as described [7]. Thus, FcεRI^+ ^expression on HuMC was downregulated by the presence of LPS, while PGN in the presence of 10 ng/ml rhSCF again inhibited HuMC numbers. Due to the inhibition of HuMC growth and development by TLR2 agonists PGN and LTA, the remaining experiments focused on LPS and its HuMC growth and survival enhancing effects.

### Effect of LPS on 8 wk old HuMC β-hex, PGD_2 _and LTC_4 _release in short-term cultures

We next proceeded to characterize the effect of LPS over 72 hr on HuMC, which had been in culture for 8 wks, as to histamine content; and β-hex, PGD_2 _and LTC_4 _release. In the presence of 10 ng/ml rhSCF, the addition of 100 ng/ml LPS increased the histamine content of HuMC over 72 hrs from a mean of 7.83 ± 2.33 pg/cell to 12.75 ± 1.94 pg/cell (n = 3, p < 0.05). We next assayed release and found that the addition of 100 ng/ml LPS to 10 ng/ml rhSCF over 72 hrs decreased β-hex release following FcεRI crosslinking from 12.21 ± 0.31% to 3.56 ± 1.35% (n = 3, p < 0.01). Following FcεRI crosslinking, LPS did not alter PGD_2 _and LTC_4 _release (13.48 ± 0.52 ng/ml and 11.26 ± 4.75 ng/ml, respectively). Thus, the addition of LPS to HuMC cultured over 72 hr with 10 ng/ml rhSCF reduced % β-hex release, but had no effect on PGD_2 _and LTC_4 _release.

### Effect of LPS on 8 wk HuMC protease expression and composition in short-term cultures

**Figure 3:**
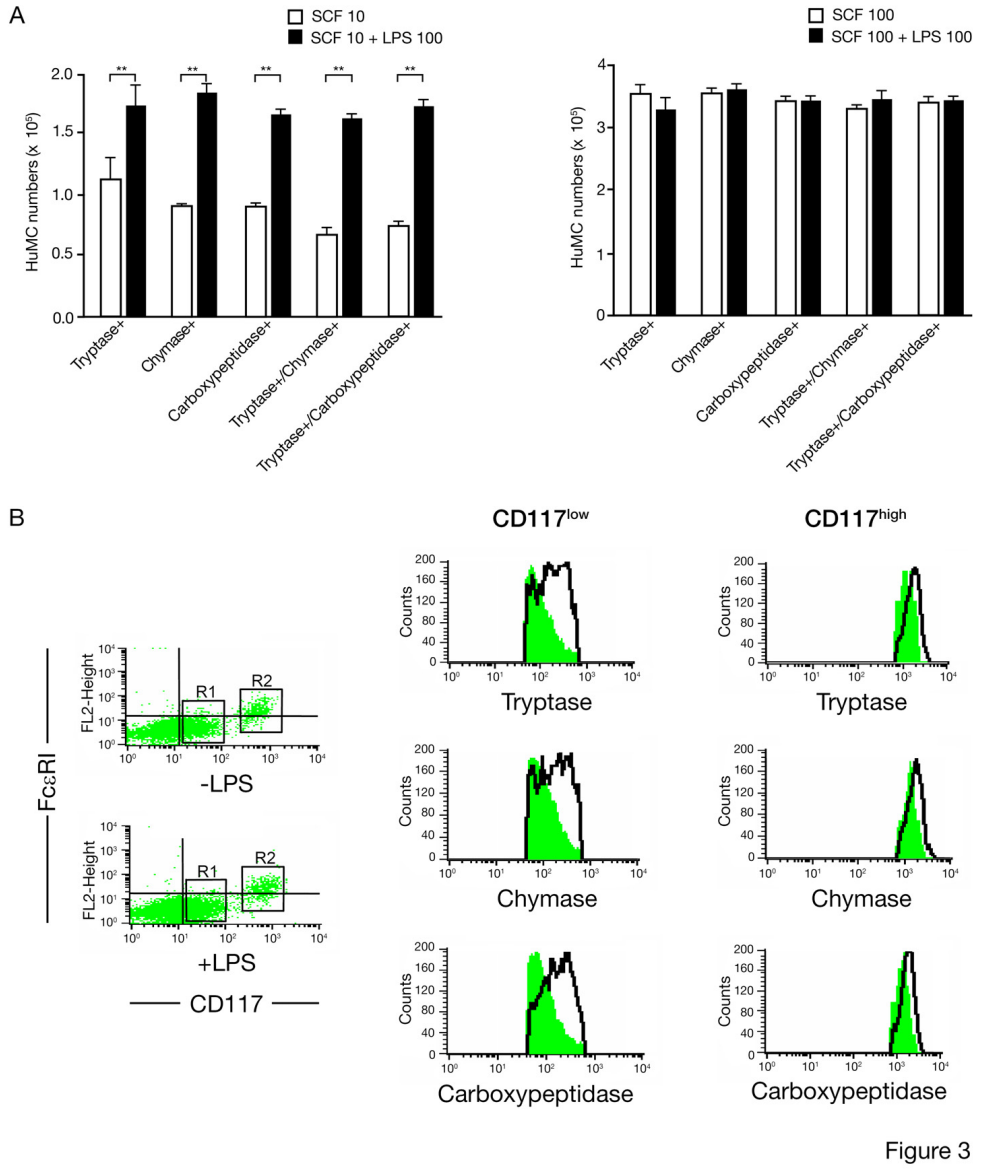
**Tryptase, chymase and carboxypeptidase expression of 8 wk HuMC cultured in rhSCF alone and with 100 ng/ml LPS; Live gate analysis of CD117^low ^(R1 region) and CD117^high ^(R2 region) cells**. A) Left-sided graph – HuMC numbers expressing tryptase, chymase, and carboxypeptidase following culture for 72 hr in 10 ng/ml rhSCF alone, and with 100 ng/ml LPS. For 10 ng/ml rhSCF alone, the mean ± SEM of percentages of protease positive HuMC are: tryptase 88.51 ± 5.30%, chymase 91.34 ± 1.62%, carboxypeptidase 91.53 ± 1.20%, tryptase/chymase 83.75 ± 4.61% and tryptase/carboxypeptidase 87.32 ± 4.90%. For 10 ng/ml rhSCF and LPS, the mean ± SEM of percentages of protease positive HuMC cells are: tryptase 92.50 ± 2.32%, chymase 96.30 ± 0.75%, carboxypeptidase 94.43 ± 4.21%, tryptase/chymase 92.33 ± 3.85%, and tryptase/carboxypeptidase 90.50 ± 2.30%. Right-sided graph – 100 ng/ml rhSCF alone, and with 100 ng/ml LPS. For 100 ng/ml rhSCF alone, the mean ± SEM of percentages of protease positive HuMC are: tryptase 94.56 ± 2.30%, chymase 93.30 ± 1.38%, carboxypeptidase 88.50 ± 3.21%, tryptase/chymase 88.70 ± 5.41%, and tryptase/carboxypeptidase 88.55 ± 3.83%. For 100 ng/ml rhSCF and LPS, the mean ± SEM of percentages of protease positive HuMC are: tryptase 92.57 ± 3.30%, chymase 95.33 ± 0.85%, carboxypeptidase 89.81 ± 3.90%, tryptase/chymase 93.63 ± 3.25%, and tryptase/carboxypeptidase 89.50 ± 5.42%. Results are shown as the mean ± SEM of 3 separate experiments performed in duplicate; B) Representative dot plots and histograms of CD117^low ^(R1 region) and CD117^high ^(R2 region) cells analyzed for FcεRI, CD117, tryptase, chymase and carboxypeptidase expression during culture in 10 ng/ml rhSCF alone and with 100 ng/ml LPS (as seen in Figure A, left). In the histograms, horizontal axis represents PE or PE5.5-conjugated markers analyzed; vertical axis represents total cell numbers analyzed. Cultures with 10 ng/ml rhSCF alone are shown by the shaded areas. Representative dot plots and histograms are shown of n = 3 separate experiments performed in duplicate.

We next examined the effect of LPS on 8 wk old HuMC protease expression and composition over 72 hrs. In the presence of 10 ng/ml rhSCF and 100 ng/ml LPS, FACS analysis showed significant increases in tryptase+, chymase+, carboxypeptidase+, tryptase+/chymase+ and tryptase+/carboxypeptidase+ HuMCs (n = 3, p < 0.01) (Fig. 3A, left) compared to cultures with 10 ng/ml rhSCF only. This effect was not observed when cells were cultured in 100 ng/ml of rhSCF (Fig. 3A; compare right with left). As shown in Figure 3B, live gate analysis of the CD117^low ^(region R1) and CD117^high ^(region R2) HuMC subpopulations, cultured in the presence of 10 ng/ml rhSCF with and without 100 ng/ml LPS, determined that increases in chymase and carboxypeptidase staining were detected predominately for the CD117^low^(region R1) subpopulation.

### Effect of LPS and PGN on 8 wk old HuMC cytokine release and the effect of cytokines on protease expression

**Figure 4:**
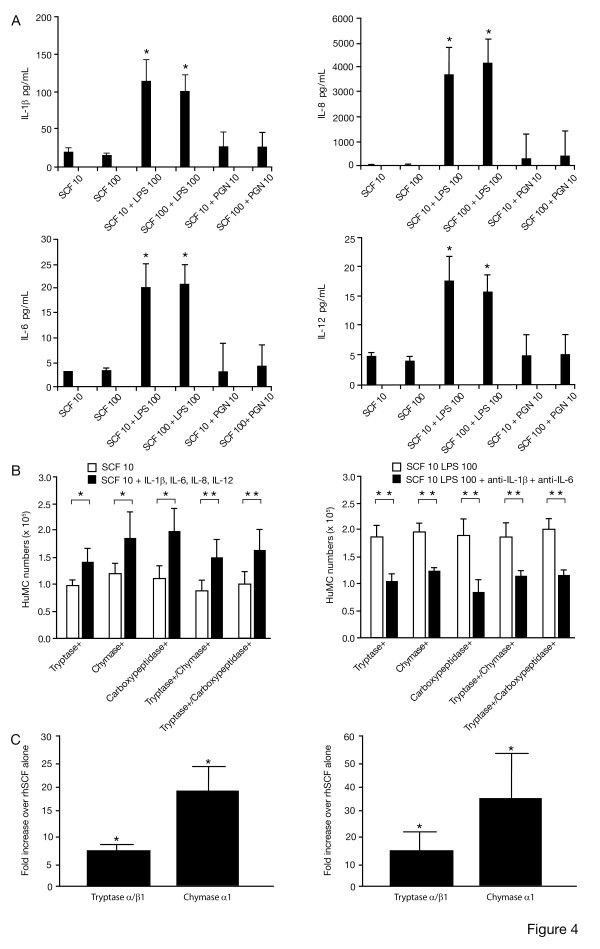
**Immunoassay of cytokine release and cytokine effect on tryptase, chymase, and carboxypeptidase expression of 8 wk HuMC by FACS and QPCR. Effect of rhIL-1β and rhIL-6 blocking antibodies**. A) Immunoassay for the presence of IL-1β, IL-6, IL-8, IL-12 and SCF in supernatants of 8 wk HuMC treated for 72 hr with rhSCF, rhSCF and LPS, and rhSCF and PGN; B) Left-sided graph – HuMC numbers expressing tryptase, chymase, and carboxypeptidase following 72 hr in culture with 10 ng/ml rhSCF alone, and with 150 pg/ml rhIL1-β, 25 pg/ml rhIL-6, 5000 pg/ml rhIL-8, and 25 pg/ml rhIL-12. For 10 ng/ml rhSCF alone, the mean ± SEM of percentages of protease positive HuMC are: tryptase 91.50 ± 3.13%, chymase 92.20 ± 3.11%, carboxypeptidase 95.73 ± 1.86%, tryptase/chymase 87.32 ± 3.76% and tryptase/carboxypeptidase 85.70 ± 4.77%. For 10 ng/ml rhSCF and rhIL1-β, rhIL-6, rhIL-8, and rhIL-12, the mean ± SEM of percentages of protease positive HuMC are: tryptase 94.99 ± 0.83%, chymase 97.23 ± 0.35%, carboxypeptidase 97.62 ± 0.50%, tryptase/chymase 89.93 ± 3.87% and tryptase/carboxypeptidase 89.80 ± 4.23%. Results are shown as the mean ± SEM of 3 separate experiments performed in duplicate. Right-sided graph – HuMC numbers expressing tryptase, chymase, and carboxypeptidase following 72 hr in culture with 10 ng/ml rhSCF and 100 ng/ml LPS, and with 0.5 μg/ml anti-rhIL-1β and 1 μg/ml anti-rhIL-6. Goat blocking antibodies are matched with goat isotype IgG control. For 10 ng/ml rhSCF and LPS, the mean ± SEM of percentages of protease positive HuMC are: tryptase 92.30 ± 1.30%, chymase 98.22 ± 0.35%, carboxypeptidase 95.53 ± 1.22%, tryptase/chymase 91.40 ± 2.77%, and tryptase/carboxypeptidase 91.43 ± 1.91%. For 10 ng/ml rhSCF, LPS, anti-rhIL-1β and anti-rhIL-6, the mean ± SEM of percentages of protease positive HuMC are: tryptase 90.60 ± 1.37%, chymase 94.71 ± 0.45%, carboxypeptidase 92.12 ± 1.22%, tryptase/chymase 88.30 ± 2.31%, and tryptase/carboxypeptidase 87.11 ± 3.05%. Results are shown as the mean ± SEM of 2 separate experiments performed in duplicate. C) Left-sided graph – QPCR for tryptase and chymase expression of HuMC cultured with rhSCF alone, and with IL-1β, IL-6, IL-8 and IL-12; Right-sided graph – QPCR for tryptase and chymase expression of HuMC cultured with rhSCF alone, and with LPS. Results are shown as the fold increase over rhSCF alone, and are the mean ± SEM of n = 3 separate experiments performed in triplicate.

Since mature 8 wk HuMC cultures do not contain other cell types, we next asked whether the effect of LPS on protease expression might be induced through endogenous HuMC cytokine release. To this end, cell culture supernatants harvested from HuMC cultures following 72 hr incubation with 10 or 100 ng/ml rhSCF alone, or with 100 ng/ml LPS were assayed for the presence of IL-1β, IL-2, IL-4, IL-6, IL-8, IL-10, IL-12, IFN-γ, SCF and TNF-α. As shown in Figure 4A, 100 ng/ml LPS induced HuMC to produce IL-1β, IL-6, IL-8 and IL-12, with no differences noted using 10 or 100 ng/ml rhSCF (n = 3, p < 0.05). No elevations in IL-2, IL-4, IL-10, IFN-γ and TNF-α were noted in the presence of LPS. As expected, SCF was detected in cultures containing SCF and LPS, but levels were not elevated above those measured in cultures with SCF alone. In addition, no elevations in cytokines were noted in cultures to which PGN had been added.

To determine if observed increases in tryptase, chymase and carboxypeptidase expression could be explained on the basis of endogenous IL-1β, IL-6, IL-8 and IL-12 release, HuMC were cultured with rhSCF 10 or 100 ng/ml alone or with rhIL-1β, rhIL-6, rhIL-8 and rhIL-12 individually or as a four-cytokine mixture, using concentrations of cytokines detected by immunoassay in the culture supernatants (Fig. 4B). The combination of 10 ng/ml rhSCF with either IL-1β or IL-6 gave rise to significant increases in tryptase+, chymase+, tryptase+/chymase+ and tryptase+/carboxypeptidase+ HuMCs, in contrast to rhIL-8 or rhIL-12 which did not increase tryptase, chymase and carboxypeptidase expression (data not shown). No increases in protease expression were observed in the presence of 100 ng/ml rhSCF with other cytokines. A combination of 10 ng/ml rhSCF with 150 pg/ml rhIL-1β, 25 pg/ml rhIL-6, 5000 pg/ml rhIL-8 and 25 pg/ml rhIL-12, cytokine concentrations detected in the cell cultures supernatant following 72 hr of incubation with 100 ng/ml LPS, gave rise to increased tryptase+, chymase+, carboxypeptidase+ (n = 3, p < 0.01), tryptase+/chymase+ and tryptase+/carboxypeptidase+ (n = 3, p < 0.05) HuMC numbers, reproducing the observed increases seen with 100 ng/ml LPS and 10 ng/ml SCF (Fig. 4B, left-sided graph). Furthermore, this effect of LPS could be significantly reduced when HuMC were cultured for 72 hr in the added presence of 0.5 μg/ml anti-IL-1β and 1.0 μg/ml anti-IL-6 antibodies (Fig. 4B, right-sided graph). QPCR analysis of HuMC cells showed an overall 8-fold increase in tryptase and 20-fold increase in chymase expression, following stimulation for 72 hr with 10 ng/ml rhSCF, 150 pg/ml rhIL-1β, 25 pg/ml rhIL-6, 5000 pg/ml rhIL-8 and 25 pg/ml rhIL-12 (Fig. 4C). Similar increases in tryptase and chymase expression were noted following stimulation for 72 hr with 10 ng/ml rhSCF and 100 ng/ml LPS (Fig. 4C, right-sided graph). Together, this data is consistent with the conclusion that LPS stimulates HuMC in culture to release IL-1β, IL-6, IL-8 and IL-12, and the overall net effect is to increase CD117^low ^HuMC production of tryptase, chymase and carboxypeptidase.

### Effect of LPS on 4–6 wk old WT and TLR4 KO BMMC protease mRNA expression in short-term culture

**Figure 5:**
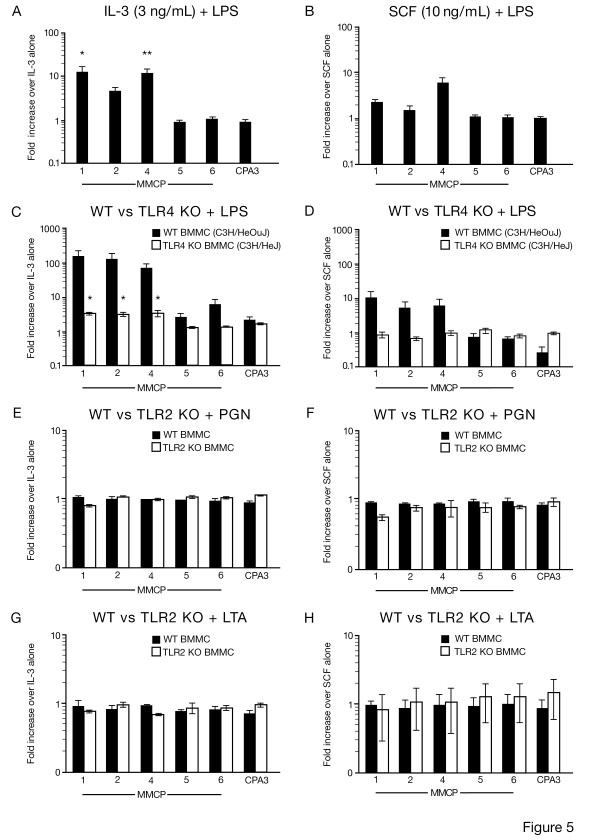
**Protease mRNA expression by WT, TLR4 KO and TLR2 KO BMMC in the presence of LPS, PGN or LTA and either rmIL-3 or rmSCF**. MMCP-1 [1], MMCP-2 [2], MMCP-4 [4], MMCP-5 [5], MMCP-6 [6] and carboxypeptidase A (CPA3) were analyzed by real-time PCR from 4–6 wk WT (C57BL/6) BMMC (A-B), WT (C3H/HeOuJ), TLR4 KO (C3H/HeJ) BMMC (C-D), and WT, TLR2 KO BMMC (E-H). BMMC were cultured for 72 hr with either 3 ng/ml rmIL-3 (A, C, E, G) or 10 ng/ml rmSCF (B, D, F, H) in the absence or presence of 100 ng/ml LPS, 10 μg/ml PGN or 1 μg/ml LTA. The effect of LPS, PGN or LTA on protease mRNA expression was determined by calculating the fold increase compared to untreated control (C57BL/6 or C3H/HeOuJ) samples. Results shown are mean ± SEM, n = 4 in triplicate for WT BMMC (A-B), n = 3 in triplicate for WT and TLR4 KO BMMC (C-D), and n = 2 in triplicate for WT and TLR2 KO BMMC (E-H).

Since short-term exposure to LPS increased protease expression in HuMC, we asked whether short-term exposure to LPS could also effect the protease mRNA expression of BMMC from WT (C57BL/6) mice. Thus, BMMC were first allowed to mature over 4–6 wks, washed and cultured in either 3 or 30 ng/ml rmIL-3, 10 or 50 ng/ml rmSCF alone and with 100 ng/ml LPS for 72 hr. BMMC were then harvested and examined by QPCR for MMCP mRNA expression. WT BMMC had constitutive mRNA expression for the proteases MMCP-4, -5, -6 and CPA3 when cultured in either rmIL-3 (3–30 ng/ml) or rmSCF (10–50 ng/ml) for 72 hr (data not shown). As shown in Figure 5, following the addition of LPS for 72 hr, an upregulation in MMCP-1, MMCP-2 and MMCP-4 mRNA expression was observed in the presence of either rmIL3 (3 ng/ml) (Fig. 5A) or in the presence of rmSCF (10 ng/ml) (Fig. 5B). A similar pattern of MMCP upregulation was also observed at the higher concentrations of rmIL3 (30 ng/ml) or rmSCF (50 ng/ml) in the presence of LPS (data not shown). Since TLR4 deficient mice are hyporesponsive to lipopolysaccharide [8], and to determine if upregulated MMCP levels by LPS were mediated by TLR4, we obtained BMMC from WT C3H/HeOuJ and TLR4 KO C3H/HeJ mice and exposed them to low concentrations of cytokine in combination with LPS. As can be seen, in the presence of either rmIL-3 (3 ng/ml) (Fig. 5C) or rmSCF (10 ng/ml) (Fig. 5D), WT BMMC from C3H/HeOuJ mice had a similar MMCP mRNA profile in response to LPS as WT BMMC from C57BL/6 mice. However, in contrast with C3H/HeOuJ, BMMC cultured from C3H/HeJ mice and incubated with mIL-3 (3 ng/ml) (Fig. 5C; compare with 5A), or rmSCF (10 ng/ml) (Fig. 5D; compare with 5B) had reduced MMCP-1, MMCP-2 and MMCP-4 mRNA expression following incubation with LPS. BMMC from both WT and TLR2 KO mice were also cultured in the presence of rmIL-3 (3 ng/ml) or rmSCF (10 ng/ml) with PGN or LTA for 72 hr, and examined for any effect on protease mRNA expression. As shown in Figures 5E–H, neither TLR2 ligand affected MMCP expression in WT or TLR2 KO mice. Taken together these data suggest that LPS, but not the TLR2 ligands PGN or LTA, induce the upregulation of MMCP-1, -2 and -4 mRNA expression in BMMC through cytokines which are produced by mast cell activation through TLR4.

## Discussion

In this report, we demonstrate that LPS and PGN have very different and specific effects on progenitor and mature HuMC. These observations are relevant, since HuMC maturation may occur in proximity to tissues such as the lung and gastrointestinal mucosa that interface with the external environment, where HuMC progenitors and their mature counterparts may variably be exposed to bacterial-derived products over time. It has been shown, for example, that inhalation of aerosolized LPS exacerbates allergic airway inflammation in the mouse experimental asthma model through TLR4-mediated mast cell activation and promotion of T_H_2 responses [9]. Cell transfer experiments using TLR4 KO mast cells confirmed that the effect of LPS is mediated through TLR4 on mast cells, consistent with our data. We also found that HuMCs in the presence of LPS synthesized de novo IL-1β and IL-12, in addition to IL-6 and IL-8, which is consistent with the induction of T_H_1 effector responses reported for mouse dendritic cell (DC) cultures [10]. If the hypothesis put forward that, under experimental conditions, toll-mediated events facilitate the generation of T_H_1 responses [10], then mouse and human *in vitro *mast cell data are in agreement, and *in vivo *inhalation of aerosolized LPS might induce both HuMC and DC to produce increased cytokine levels that lead to the promotion of T_H_1-skewed responses. The net effect of these T_H_1 responses in combination with LPS-induced T_H_2 responses would determine the overall extent of allergic airway inflammation.

We thus examined CD34^+^-derived HuMC progenitors over 6 wks (long-term cultures) and 8 wk mature HuMC over 72 hr (short-term cultures) for the effect of LPS, PGN and LTA on cell numbers; and CD117, FcεRI, cytokine, tryptase, chymase and carboxypeptidase expression. In continuous culture over 6 wks, LPS had little or no effect on HuMC numbers, while PGN led to a decrease in HuMC numbers at all concentrations (Fig. 1). LTA, another TLR2 agonist, also decreased HuMC numbers at the highest concentrations. LPS, but not PGN, led to small increases in CD117, tryptase and chymase expression compared with rhSCF alone, while decreasing FcεRI expression (data not shown).

These differential effects of LPS and PGN became more evident when mature 8 wk HuMC were used in short-term cultures. When 8 wk HuMC, originating from CD34^+ ^cells in rhSCF, were cultured with PGN and 10 ng/ml rhSCF for 72 hr, PGN reduced HuMC numbers below those observed with 10 ng/ml rhSCF alone. PGN had no effect on HuMC numbers when 100 ng/ml rhSCF was used. LPS somewhat enhanced HuMC numbers in the presence of 10 ng/ml rhSCF. FcεRI expression was again reduced in cultures containing either 10 or 100 ng/ml rhSCF and LPS (Fig. 2), consistent with the observation that the LTA, following ligation with TLR2, reduced surface expression of FcεRI in isolated human pulmonary mast cells [11]. In the presence of 10 ng/ml rhSCF, the reduction in FcεRI expression could be seen to affect two CD117 subpopulations: CD117^low ^and CD117^high ^HuMCs (Fig. 2B). The MFI of the CD117^high ^cells increased a log-fold higher, suggesting that 100 ng/ml of rhSCF suppressed CD117 expression on HuMC, consistent with other reports [12]. In the presence of LPS and 10 ng/ml, but not 100 ng/ml of rhSCF, tryptase positive HuMC co-expressing chymase and carboxypeptidase increased (Fig. 3A), and live gate analysis showed that increases in tryptase, chymase and carboxypeptidase occurred predominately in the CD117^low ^subpopulation (Fig. 3B). These data therefore demonstrate that short-term exposure of mature HuMC to varying concentrations of rhSCF alters CD117 expression [12], and protease expression; and that these effects are modulated by LPS. Of note is that 100 ng/ml rhSCF that we and others use for optimal HuMC in vitro growth may mask the upregulatory effects of LPS, when LPS is added to cultures.

It has been reported that activation of murine mast [13,14] and dendritic [15] cells through TLRs augments production of inflammatory cytokines, and that cytokines can influence human mast cell protease expression [16,17]. To determine whether LPS-driven short-term cultures induced HuMC-derived cytokine production which in turn could influence cell survival as well as changes in surface receptor and protease expression, we assayed short-term cultures and found, as noted above, significant levels of IL-1β, IL-6, IL-8 and IL-12 in LPS-driven, but not PGN-driven, cultures. Furthermore, we were able to reproduce the effects of LPS on HuMC by culturing these cells in 10 ng/ml rhSCF and immunoassay-detected concentrations of IL-1β, IL-6, IL-8 and IL-12 (Fig. 4B). The combination of 10 ng/ml rhSCF with either IL-1β or IL-6, but not IL-8 or IL-12, also reproduced the LPS observed effects in short-term cultures. Thus, one possible mechanism of action of LPS is to stimulate production of mature HuMC-derived IL-1β and IL-6, which together promote HuMC survival, alter HuMC FcεRI and protease expression, and together with IL-12 direct HuMC involvement in resolving innate immunity and promoting acquired immune responses. This transition from innate to acquired immunity involves the resolution of an inflammatory event, and as demonstrated in mouse models, IL-6 is involved in reducing bacteria-induced inflammation [18-20]. In addition, although IL-8 was not shown to affect HuMC survival, surface receptor and protease expression, IL-8 production by HuMC might in part explain how LPS can promote neutrophil influx into inflammatory sites and promote host defense mechanisms.

The conclusion that LPS upregulates HuMC protease expression and composition were further supported using BMMC from WT and TLR4 KO mice (Fig. 5). It is known that exposure of WT BMMC to rmIL3 and rmSCF alters the protease composition of these cells [21]. Following exposure of WT BMMC to reduced concentrations of rmIL-3 or rmSCF in the presence of LPS an increase in mRNA for the MMCP-1, -2 and -4 was observed. BMMC from TLR4 KO mice, following stimulation with LPS, failed to show any alteration in MMCP-1, MMCP-2, and MMCP-4 mRNA expression compared with WT controls. These findings demonstrate that the differential mRNA expression of MMCP depends on the presence and concentration of rmIL-3 or rmSCF used to culture BMMC, and that the MMCP mRNA expression of BMMC can be influenced by LPS ligation with TLR4, an effect which is eliminated in the absence of a functional TLR4 receptor. MMCP-1 mRNA expression in mucosal mast cells of Trichinella spiralis-infected mice can similarly be influenced by IL-10 [22]. Furthermore, MMCP-1, MMCP-2 and MMCP-4 are chymases, and MMCP-4 most probably represents the murine homolog of human chymase [23]. In addition, the observation that TLR2 ligands PGN and LTA had no effect on MMCP mRNA expression from WT and TLR2 KO-derived BMMC appears to be consistent with the observation in HuMC (Figure 4) that LPS, but not PGN, increases HuMC numbers and protease expression, and may be related to the LPS-induced increase in IL-1β and IL-6.

The upregulation of human chymase and carboxypeptidase by LPS may be another means by which mast cells participate in endotoxin-driven alteration in T_H_1 and T_H_2 responses. Mast cell-derived chymase and carboxypeptidase are involved in resistance to toxic endogenous or exogenous peptides such as endothelin-1 [24] and venoms [25] respectively; thus, their induction may counterbalance the effect of tryptase which is known to increase T cell-derived IL-13 and airway hyper-responsiveness [26].

## Conclusion

Taken together, these data demonstrate that LPS can alter HuMC numbers, influence cytokine and protease expression and composition, and that the effect of LPS is best observed when reduced concentrations of rhSCF are used, as may occur in tissues. These data further support the idea that the appearance of LPS during a gram negative bacterial infection, in the presence of tissue concentrations of SCF, may induce tissue mast cells to express a unique composition of proteases beneficial for controlling and eliminating this particular infection. In other infections or clinical states having higher concentrations of other cytokines and/or the presence of other mediators, tissue mast cell progenitors may differentially develop and express specific surface receptors and proteases which enable these mast cells to more effectively participate in innate immune responses.

## Methods

### CD34^+ ^immunoselection

Peripheral blood (PB) mononuclear cells collected by leukapheresis were obtained from normal volunteers following informed consent [1]. CD34^+ ^progenitor cells were enriched to 95–99% by positive immunomagnetic selection or using commercially available affinity columns.

### HuMC long-term and short-term cultures

#### Long-term cultures

Peripheral blood CD34^+ ^cells were placed at a concentration of 5 × 10^4 ^cells/ml in serum-free media (SFM) (StemPro-34 SFM; Life Technologies, Grand Island, NY) supplemented with 2 mM L-glutamine, 100 IU/ml penicillin, 50 μg/ml streptomycin (complete SFM) and 100 ng/ml recombinant human stem cell factor (rhSCF) (Peprotech, Rocky Hill, NJ) alone or in combination with either 10–1000 ng/ml LPS (Sigma-Aldrich, St. Louis, MO), 10–1000 μg/ml PGN (Fluka, Ronkonkoma, NY) or 0.1–10 μg/ml LTA (Invivogen, San Diego, CA). Heat inactivated human serum was added to cultures containing LPS. Cells were counted and stained weekly with Kimura's stain and tested for tryptase positivity to determine total cell and HuMC numbers as described [1,27]. Kimura's and tryptase staining of HuMC did not differ by greater than 5%. Cytokines and TLR ligands were replaced weekly. *Short term cultures*: Eight wk HuMC were cultured in 100 ng/ml rhSCF, washed and then incubated for 72 hr with either 10 or 100 ng/ml rhSCF alone, or in combination with either 100 ng/ml LPS or 10 μg/ml PGN. In some experiments, 8 wk HuMC were cultured with cytokines (IL-1β, IL-6, IL-8, or IL-12) alone or in combination (Peprotech, Rocky Hill, NJ), in addition to blocking antibodies for IL-1β (R&D, Minneapolis, MN) and IL-6 (Peprotech, Rocky Hill, NJ). Cells were harvested and prepared for flow cytometry, mediator and cytokine release, and real-time PCR for analysis of protease expression.

### BMMC from WT and TLR4 KO mice

WT (C57BL/6, C3H/HeOuJ), TLR4 KO (C3H/HeJ) and TLR2 KO mice (< 6 months old, 20 g) were obtained from Jackson Laboratories (Bar Harbor, ME). Mouse bone marrow-derived mast cells (BMMC) were cultured from femoral marrow cells in RPMI 1640 medium supplemented with 10% FBS, 100 U/ml penicillin, 100 μg/ml streptomycin, 25 mM HEPES, 1.0 mM sodium pyruvate, non-essential amino acids, 0.0035% 2-mercaptoethanol (complete RPMI) and 30 ng/ml recombinant mouse (rm) IL-3 (Peprotech, Rocky Hill, NJ). In all experiments, 4–6 wk old BMMC were harvested, washed in complete RPMI, and incubated with 10–50 ng/ml rmSCF (Peprotech, Rocky Hill, NJ), or 3–30 ng/ml rmIL-3 alone or in combination with 100 ng/ml LPS, 10 μg/ml PGN or 1 μg/ml LTA.

### β-hexosaminidase (β-hex) release

HuMC were sensitized overnight with 100 ng/ml of biotinylated human IgE (biotIgE). Cells were then washed to remove excess biotIgE, and crosslinked using streptavidin (SA) (BD PharMingen, San Diego, CA) as previously described [28]. *β*-hex was reported as % release. %β-hex release after 72 hr was calculated as net relative to the non-activated cells in the presence of SCF+LPS and compared with HuMC treated with SCF alone.

### Histamine, LTC_4 _and PGD_2 _assay

Cellular histamine content and LTC_4 _and PGD_2 _release were examined in duplicate using 1,000–10,000 HuMC washed in HEPES with 0.04% BSA. Histamine content was analyzed with a commercial histamine ELISA Kit (Immunotech, Cedex, France) and reported as pg/cell. LTC_4 _and PGD_2_release were analyzed by competitive enzyme immunoassay (EIA) (Cayman Chemicals, Ann Arbor, MI) and reported as ng/ml.

### Flow cytometric analysis

HuMC surface marker and intracytoplasmic staining was performed using a modified approach for mononuclear cells [29]. Briefly, cells were incubated sequentially with one or more of the following conjugated antibodies: 5 μg/ml biotIgE; 2.5 μg/ml R-phycoerythrin (PE) (1 mg/ml; Ancell, Bayport, MN) or allophycocyanine (APC)-conjugated CD117 (Becton-Dickinson, San Jose, CA), 2.0 μg/ml PE5.5 (Caltag/Invitrogen, Carlsbad, CA) or 2.5 μg/ml APC-conjugated streptavidin (SA) (0.2 mg/ml; BD PharMingen, San Diego, CA) per final volume of 50 μl containing 20,000–50,000 nucleated cells). PE conjugated annexin V was used to determine apoptosis (Detection Kit I; BD PharMingen, San Diego, Ca). In all experiments, 10,000–20,000 cell events were analyzed. Cell analysis was performed using a FACScan and CellQuest software (Becton Dickinson, San Jose, CA).

### Intracytoplasmic staining for tryptase, chymase and carboxypeptidase

HuMCs were fixed and permeabilized using 4% paraformaldehyde and 1 × PBS with 0.1% saponin and stained for tryptase, chymase and carboxypeptidase as described [7,29]. For tryptase/chymase or tryptase/carboxypeptidase staining, cells were first stained for tryptase using 3 μg/mL mouse antihuman tryptase (Clone G3, 1.14 mg/mL, Chemicon, Temecula, CA) and 5.0 μg/ml PE-conjugated goat antimouse IgG (0.5 mg/ml, Southern Biotechnology, Birmingham, AL). Cells were washed and incubated with either 3 μg/mL biotinylated mouse antihuman chymase (Clone B7, 3 mg/mL, Chemicon, Temecula, CA) or 3 μg/ml biotinylated mouse antihuman carboxypeptidase (kind gift of Dr. Andrew Walls, University of Southampton, UK). Cells were washed and incubated with 2.5 μg/ml APC-conjugated SA. Cell analysis was performed using 20,000–50,000 nucleated cells per final volume of 50 μl. In all experiments, 10,000–20,000 cell events were analyzed using a FACScan and CellQuest software. In all experiments, the numbers of HuMC positive for one or more proteases was calculated by multiplying the total number of cells examined in culture by the percentage of gated, single or dual protease positive cells determined using flow cytometry.

### Human cytokine immunoassay

Quantitative enzyme immunoassays for IL-1β, IL-2, IL-4, IL-6, IL-8, IL-10, IL-12 (measured as the heterodimer IL-12p70), IFN-γ, SCF and TNF-α in cell-free culture supernatants were performed using the following commercial cytometric bead array (CBA) kits; Human Chemokine Kit and Human Th1/Th2 Cytokine Kit II (BD Biosciences, San Jose, CA) and analyzed on a FACSArray Bioanalyzer. The minimum detection levels for the chemokines and cytokines are IL-1β, 7.2 pg/mL, IL-2, 2.6 pg/mL, IL-4, 2.6 pg/mL, IL-6, 3.0 pg/mL, IL-8, 0.2 pg/mL, IL-10, 2.8 pg/mL, IL-12, 1.9 pg/mL, IFN-γ, 7.1 pg/mL and TNF, 2.8 pg/mL.

### Real-time PCR analysis for HuMC and BMMC protease mRNA expression

Total RNA was isolated from each preparation using the RNeasy Mini Kit (Qiagen, Valencia, CA). For HuMC, one microgram of total cellular RNA was treated for genomic DNA contamination and reverse transcribed using Invitrogen Reverse Transcription reagents and oligo dT. Gene expression was analyzed using real-time PCR on an ABI7500 SDS system. One hundred ng of cDNA was used in each quantitative PCR assay, and tryptase and chymase primer sets (Qiagen, Valencia, CA) for PCR amplifications were designed using Primer Express software (PerkinElmer, Shelton, CT). All reactions were performed in triplicate for 40 cycles. Samples were normalized using the geometric mean of GAPDH15 and data are reported as the ratio of treated cells/untreated control cells. Different stocks of cDNA were used for all amplifications to determine the relative quantities across multiple runs.

For BMMCs, two micrograms of total cellular RNA was treated for genomic DNA contamination and reverse transcribed using the Qiagen genomic DNA wipeout buffer and reverse transcription reagents. For each quantitative PCR assay, fifty ng of cDNA was mixed with PCR mastermix containing primer sets (Qiagen, Valencia, CA) for mouse mast cell proteases (MMCP)-1, MMCP-2, MMCP-4, MMCP-5, MMCP-6, carboxypeptidase A (CPA3) or the housekeeping gene β-actin. As a control, RNA which had not being reverse transcribed to cDNA was used in some PCR reactions. All reactions were performed in triplicate for 40 cycles, and gene expression analyzed using the Real-Time PCR cycler ABI PRISM 7700 (Applied Biosystems, Foster City, CA). The relative fold expression levels of MMCPs between treatment groups was calculated as follows; for each sample the threshold cycle (Ct) was determined and normalized to β-actin (ΔCt). The ΔCt of treated cells was then subtracted from untreated control cells (ΔΔCt) and the relative fold expression was calculated using the formula 2^ΔΔCt^. cDNA stocks were taken from 3 different cultures of BMMC to determine relative quantities across multiple runs.

### Statistical Analysis

Statistical analysis of data was performed using Student's paired t-Test. Significant differences of the means were represented as either * (p < 0.05), ** (p < 0.01) or *** (p < 0.001).

## Authors' contributions

ASK performed HuMC growth, LPS and PGN culture and flow cytometric studies; ES performed PGD_2 _and LTC_4 _analysis and BMMC WT and KO studies; MK performed cytokine analysis and protease QPCR studies; YW performed mediator release studies, and DDM critically reviewed the data and helped format the paper. All authors read and approved the final manuscript.
